# HTLV-1-associated myelopathy/tropical spastic paraplegia with sporadic late-onset nemaline myopathy: a case report

**DOI:** 10.1186/s12891-023-06461-3

**Published:** 2023-05-06

**Authors:** Eiji Matsuura, Satoshi Nozuma, Ayano Shigehisa, Mika Dozono, Tomonori Nakamura, Masakazu Tanaka, Ryuji Kubota, Akihiro Hashiguchi, Hiroshi Takashima

**Affiliations:** 1 Department of Neurology and Geriatrics, Graduate School of Medical and Dental Sciences, Kagoshima University, Sakuragaoka 8-35-1, Kagoshima, 890-8544 Japan; 2 Division of Neuroimmunology, Joint Research Center for Human Retrovirus Infection, Kagoshima University, Sakuragaoka 8-35-1, Kagoshima, 890-8544 Japan

**Keywords:** SLONM, HTLV-1, HAM, TSP, Nemaline myopathy

## Abstract

**Background:**

Sporadic late onset nemaline myopathy (SLONM) is a muscle disorder characterized by the presence of nemaline rods in muscle fibers. SLONM has no known genetic cause but has been associated with monoclonal gammopathy of undetermined significance and with human immunodeficiency virus (HIV) infection. Human T-cell leukemia virus-1 (HTLV-1) is a known causative agent of adult T-cell leukemia/lymphoma and HTLV-1 associated myelopathy/tropical spastic paraplegia (HAM/TSP), a chronic inflammatory neurological disease. HTLV-1 has been reported to be implicated in inflammatory myopathies, as well as in HIV infection.; however, there have been no reports of an association between HTLV-1 infection and SLONM to date.

**Case presentation:**

A 70-year-old Japanese woman presented with gait disturbance, lumbar kyphosis, and respiratory dysfunction. The diagnosis of HAM/TSP with SLONM was made based on characteristic clinical symptoms of HAM/TSP, such as spasticity in the lower extremities, and cerebrospinal fluid test results; and of SLONM, such as generalized head drooping, respiratory failure, and muscle biopsy results. Steroid treatment was initiated and improvement in her stooped posture was observed after 3 days of treatment.

**Conclusion:**

This is the first case report of SLONM combined with HTLV-1 infection. Further studies are needed to elucidate the relationship between retroviruses and muscle diseases.

## Background

Nemaline myopathy is a disorder characterized by the presence of nemaline rods in muscle fibers and is a slowly progressive genetic muscle disease typically diagnosed in childhood. In contrast, sporadic late onset nemaline myopathy (SLONM) has no known associations with genetic mutations, develops subacutely in adults aged 40 years and older, and can involve weakness in the neck muscles (head drop) and respiratory dysfunction. The presence of nemaline rods on muscle biopsy is essential for the diagnosis of both conditions. SLONM has been reported to be associated with monoclonal gammopathy of undetermined significance (MGUS) [1, 2], systemic lupus erythematosus, myasthenia gravis, Sjögren's syndrome [3–5], and other immune-mediated diseases, while SLONM has been associated with human immunodeficiency virus (HIV), a retrovirus [6–8]. Immunomodulatory therapies, such as immunosuppressive agents and autologous peripheral blood stem cell transplantation after high-dose melphalan administration, are effective treatments for patients with SLONM-MGUS [9, 10], whereas high-dose intravenous methylprednisolone and intravenous immunoglobulin therapy is effective for SLONM without MGUS [11]. Thus, the precise cause of SLONM remains unknown.

Human T-cell leukemia virus-1 (HTLV-1) is a human retrovirus mainly transmitted from mother to child through breastfeeding. Current estimates suggest that about 10–20 million people are living with HTLV-1 infection. The majority of HTLV-1 carriers appear to remain asymptomatic throughout life, but a few percent develop HTLV-1-associated diseases, including adult T-cell leukemia/lymphoma [12], HTLV-1-associated myelopathy/tropical spastic paraparesis (HAM/TSP) [13], and HTLV-1-associated uveitis [14] between about 40 and 70 years of age. Patients with HAM/TSP present with pyramidal tract symptoms and sensory deficits in the lower body and sphincter dysfunction, while the upper body remains asymptomatic for life. SLONM has been reported to be associated with HIV infection, but, as far as we are aware, it has not been linked to HTLV-1 infection. Here, we report the first case of HAM/TSP with SLONM.

## Case presentation

**Fig. 1:**
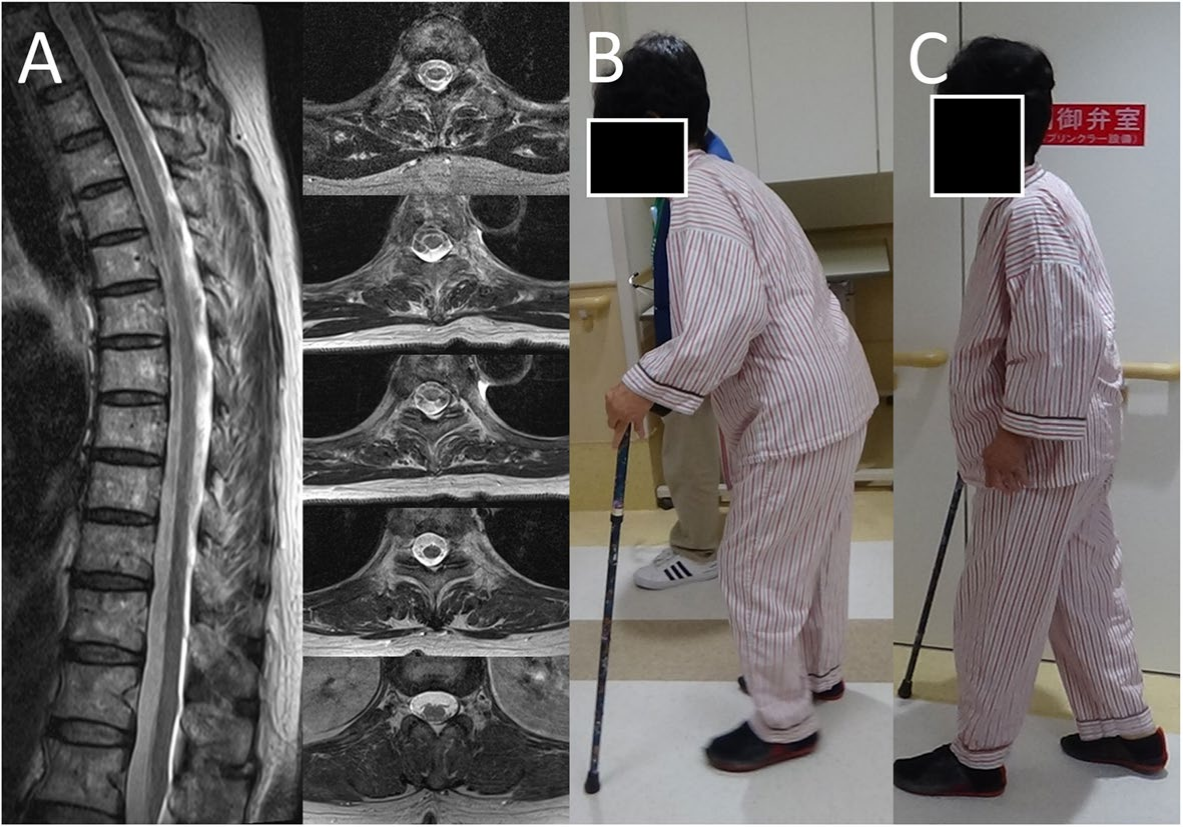
Change in patient’s walking posture before and after steroid treatment. **A** The MRI T2-weighted imaging (T2WI) revealed slight paraspinal muscle atrophy with no spinal kyphosis. Images of the patient before (**B**) and after (**C**) high-dose intravenous methylprednisolone therapy for 3 days, resulting in marked improvement of walking

A 70-year-old Japanese woman had been treated at a nearby orthopedic hospital for 3 years for low back pain and forward-leaning posture. She had a 10-year history of rheumatoid arthritis that had been treated successfully with 8 mg of methotrexate weekly, and, except for the back pain of 3 years duration, there was no swelling or pain in any of the joints in her extremities. During the preceding 1–2 years, the patient had noticed an increased frequency of falls with bilateral muscle weakness in the lower extremities. MRI and x-ray examination of the brain and entire spinal cord revealed only mild paraspinal muscular atrophy without any atlantoaxial dislocation or lumbar spinal canal stenosis (Fig. 1A). The orthopedic surgeon referred her to our department for evaluation of the difficulty in maintaining an upright posture and lower extremity muscle weakness, which was accompanied by only mild degenerative changes in the spine. The patient has a family history of certain medical conditions. Her older sister has been diagnosed with rheumatoid arthritis, and her older brother has Parkinson's disease. However, there is no history of neuromuscular disorders within the family.

On admission, the patient’s body temperature was 36.9 °C, the pulse rate was 78 beats per minute, and the blood pressure was 129/65 mmHg. She was alert, her mental status was normal, and there were no abnormalities in the head and neck region, including cranial nerve function. There were no major deformities of the extremity joints, while slight deformities of the finger joints were noted. Chest examination revealed no lung murmurs, and chest X-rays showed no abnormalities. Heart sounds and an electrocardiogram were normal. The MRI T2-weighted images (T2WI) of the spine showed no significant deformity of the thoracic or lumbar spine, no kyphosis or kyphosis, and only mild atrophy of the paraspinal muscles of the thoracic spine (Fig. 1a). The MRI Short Tau Inversion Recovery (STIR) sequence of the spine was evaluated for paraspinal muscle inflammation using sagittal sections. However, no evidence of inflammatory changes was detected within the paraspinal muscles (Data not shown). Gait examination revealed a wide-based gait with stooping posture and difficulties with tandem gait or squatting (Fig. 1B). She could not walk fast, and it took her 34 s to walk 10 m (Table 1). Symmetrical proximal muscle weakness, notably within the iliopsoas and quadriceps, was observed with Medical Research Council Grade 4/5. The other muscles of the extremities were normal. Deep tendon reflexes were enhanced bilaterally in the biceps brachii and quadriceps femoris and were normal elsewhere. Muscle tone in the upper limbs was normal, but spasticity was observed in the lower limbs. Babinski signs were detected bilaterally. Sensory examination, including deep sensation, showed no abnormalities. Cerebellar function was nearly normal in the finger–nose–finger test, although it was associated with terminal fine tremors in both hands. There was no history of autonomic nervous system symptoms such as frequent urination, constipation, or orthostatic hypotension, while skin dryness was observed from the lower abdomen to both lower extremities, which was thought to be a sweating disturbance.

**Table 1:** Evaluation of the patient’s motor function before and after steroid therapy

Examinations	Day -6 test results	Day 4 test results
10 m walk test (steps/time) 1st exam	54 steps / 34 s	34 steps / 23 s
2nd exam	50 steps / 31 s	31 steps / 23 s
2 min walk test (distance)	35 m	54 m
Manual muscle test (MMT) of iliopsoas	Right 4/5, Left 4/5	Right 5/5, Left 5/5

The patient was evaluated 6 days before receiving high-dose methylprednisolone (1000 mg per day) infusion therapy on Days 1, 2, and 3 and then re-tested on Day

Respiratory function tests showed mixed ventilatory impairment with a vital capacity of 67.5% and forced expiratory volume in 1 s of 60.61%. Because no abnormalities were detected on chest examination, trunk muscle dysfunction was suspected as the cause of ventilatory impairment. Blood gas test results were pH 7.398, pCO2 42.8 mmHg, pO2 69.9 mmHg, and HCO3 25.9 mmol/L.

Laboratory test results included erythrocyte sedimentation rate (Westergren method) 23 mm/h (normal: 3–15 mm/h), hemoglobin 12.2 g/dL, hematocrit 38.7% (mean corpuscular volume 93.9 fL, mean corpuscular hemoglobin [MCH] 29.6 pg, MCH concentration 31.5 g/dL). White blood cell count was 3980/mm3 and platelet count was 230,000/mm3. Urinalysis, liver, and renal function tests; lipid tests; and thyroid function tests were normal. C-reactive protein level was 0.75 mg/dL (normal: < 0.14 mg/dL), soluble interleukin-2 receptor was 896 U/mL (normal: 122–496 U/mL), and creatine kinase was normal. Immune function analysis showed elevated rheumatoid factor (RF) at 56.3 IU/L (normal: < 13 IU/L) and slightly elevated serum matrix metalloproteinase-3 (MMP-3) at 60.1 ng/mL (17.3–59.7 ng/mL). Levels of anti-cyclic citrullinated peptide (anti-CCP) antibody were elevated at 67.3 U/mL (normal: < 4.5 U/mL), but all other antibodies tested, including anti-nuclear, anti-dsDNA, anti-Sm, anti-RNP, anti-SS-A, and anti-SS-B antibodies, were not elevated. M-paraproteinemia was not detected. Serum was positive for anti-HTLV-1 antibody by chemiluminescence enzyme immunoassay. HTLV-1 proviral load (PVL) was 10.56 copies per 100 peripheral blood cells. An anti-HIV antibody test was negative. Anti-hepatitis B virus (HBV) core and anti-HBV surface antibody tests were positive, but HBV surface antigen and HBV DNA tests were negative.

Evaluation of cerebrospinal fluid (CSF) showed normal cell counts, protein levels, and sugar levels, but neopterin and CXCL10, two markers of inflammation in myelitis, were elevated at 49 pmol/mL (normal: 0–5 pmol/mL) and 5331.9 pg/mL (normal: 0–200 pg/mL), respectively, suggesting an ongoing inflammatory process in the central nervous system [15]. HTLV-1 antibody titer in CSF was elevated (1:1024 dilution) and HTLV-1 PVL was 20.55 copies per 100 CSF cells.

A needle electromyogram revealed a short-duration and polyphasic pattern of motor unit potentials and early recruitment pattern in the iliopsoas muscle, but without increased insertion activity or any fibrillation potential. The needle electromyogram of the tibialis anterior muscle showed no abnormalities. Nerve conduction studies performed in the left upper and lower extremities were within normal limits and F-wave latencies were also within normal limits. The F-wave frequency was > 90% in the right ulnar nerve and right tibial nerve. Somatosensory evoked potential tests performed at the bilateral tibial nerves showed slightly prolonged latencies. P40 latencies were 44.6 ms on the left side and 45.6 ms on the right side. The patient’s height was 153.9 cm. Because peripheral nerve conduction velocities were within normal limits, we concluded that the central conduction time was prolonged. Notably, prolonged central conduction time has been reported to be a possible finding in HAM/TSP [16, 17].

**Fig. 2:**
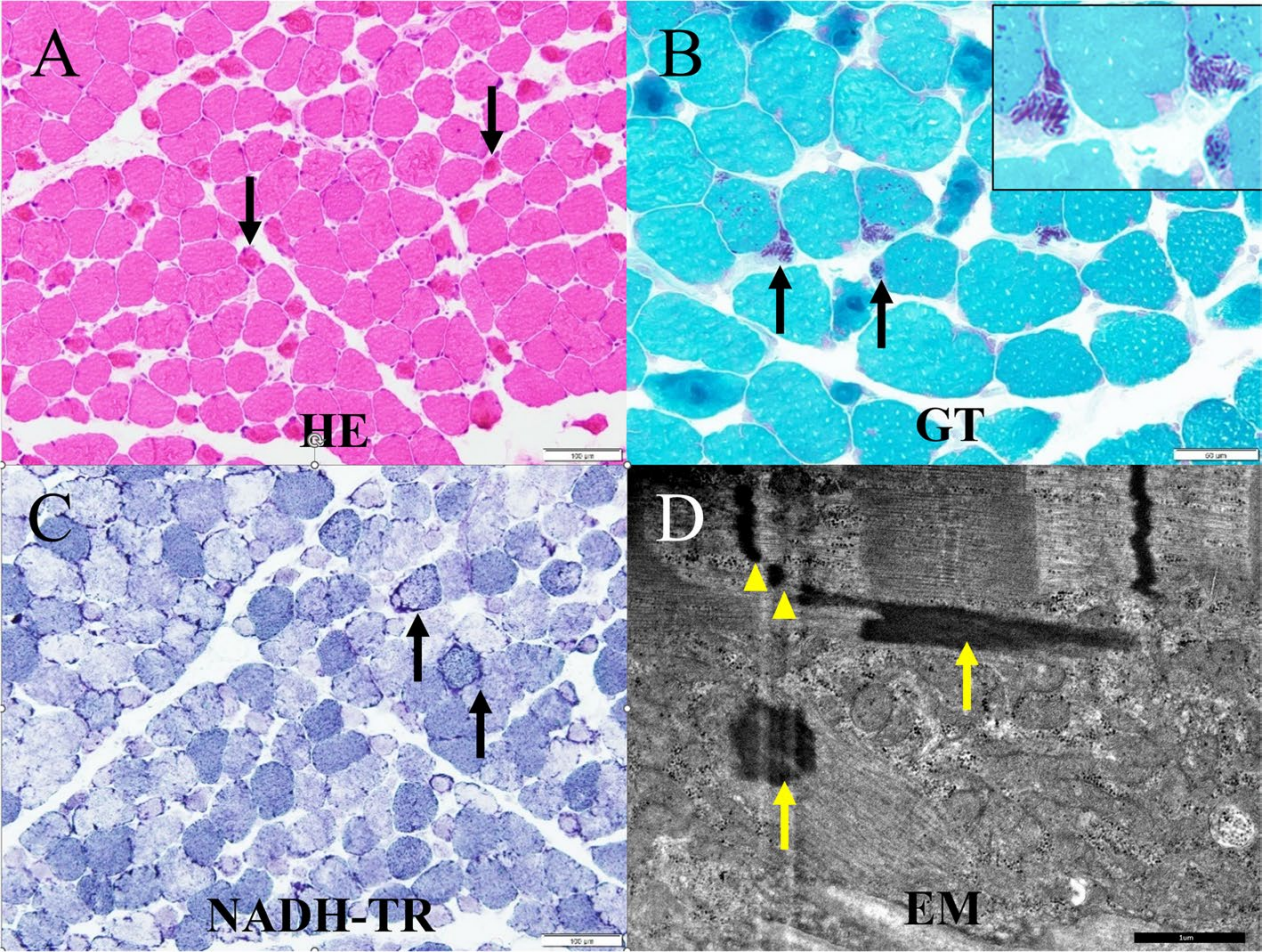
Histopathology of biopsied biceps brachii muscle. **A** Hematoxylin and eosin (HE) staining revealed many degenerating or atrophied muscle fibers (arrows) but no necrotic fibers. **B** Modified Gomori–Trichrome (GT) staining revealed nemaline rods (arrows) in some fibers and abnormal cytoplasmic agglutination (arrow heads) within atrophied muscle fibers. **C** Nicotinamide adenine dinucleotide tetrazolium reductase (NADH-TR) staining revealed a lobulated appearance (arrows) and focally increased or decreased oxidative enzyme activity. **D** Electron microscopy (EM) indicated that nemaline bodies (arrows) were of the same electron density as the Z line, with some bodies being physically connected to the Z line (arrow heads)

A pathological study was conducted on biceps brachii muscle tissue because the upper limb muscles are typically not affected in HAM/TSP. Microscopic examination of the biopsied left biceps brachii muscle revealed connective tissue elements with normal morphology (Fig. 2). Muscle fibers ranged between 15 and 70 μm in diameter and many fibers appeared to be degenerating without necrosis. Atrophied fibers with irregular sarcolemma and abnormal cytoplasm were common. Modified Gomori–Trichrome staining revealed nemaline rods; when evaluated by electron microscopy, these rods exhibited similar electron densities to those of the muscle Z lines. Likewise, some fibers had a lobulated appearance. The frequency of fibers with rods was estimated to be approximately 5.3%. Nicotinamide adenine dinucleotide tetrazolium reductase staining revealed focal areas of both decreased and increased activity. Adenosine monophosphate deaminase and cytochrome c oxidase activities were within normal limits. A random checkerboard distribution of histochemical fiber types was preserved. Acid phosphatase activity was increased slightly in some myofibers, although muscle fiber glycogen and lipid content appeared to be normal.

The patient received high-dose intravenous methylprednisolone therapy (1000 mg per day) for 3 days. Testing performed on Day 4 showed marked improvement in the strength of the iliopsoas muscles, as well as in posture and stride when walking (Fig. 1C). Notably, the time to completion and the number of steps taken in the 10-Meter Walk Test were both remarkably improved, as was the distance covered in the 2-Minute Walk Test (Table 1). Interestingly, these improvements in function were sustained for approximately 3 months after treatment, at which time the pre-treatment posture returned.

## Discussion and conclusions

We report the case of a 70-year-old Japanese woman with a stooped posture during standing and walking in addition to ventilatory disturbance. Findings of spasticity of both lower extremities with Babinski’s sign on neurological findings, loss of sweating below the abdomen, and elevated anti-HTLV-1 antibody titers in both serum and CSF met the World Health Organization criteria for the diagnosis of HAM/TSP [18]. The prolonged P40 latency found in the SEP test of the lower extremities suggested spinal cord damage [17], and elevated neopterin and CXCL10 in the CSF suggested inflammation of the central nervous system [15], both of which are common manifestations of HAM/TSP. The fact that the patient's symptoms improved markedly with steroid treatment may also suggest that the steroids ameliorated the inflammation caused by HAM/TSP. However, given that HIV-related SLONM has been reported to respond to immunotherapy [11], it is not possible to determine whether steroid treatment improved only HAM/TSP. On the other hand, respiratory dysfunction is not seen in patients with HAM/TSP, a disease that almost never affects the upper body. In SLONM patients, the frequencies of muscle weakness of the trunk and dyspnea are 68% and 55%, respectively [8]; and neck-drooping syndrome has also been reported in SLONM patients [10]. These symptoms, the presence of nemaline rods found on muscle biopsy, and the electromyography results suggestive of non-inflammatory myopathy led to the additional diagnosis of SLONM in this patient.

HIV has been suggested to be associated not only with SLONM but also with sporadic inclusion body myositis (sIBM) [19]. HTLV-1 has also been suggested to be associated with sIBM and polymyositis [19–23], though there have been no reports of its association with SLONM. In a study evaluating muscle tissue from 11 HAM/TSP patients, four showed evidence of nonspecific inflammatory myositis and three showed evidence of noninflammatory myopathy [24]. While HTLV-1 is known to infect CD4-positive lymphocytes and downregulate the expression of cell surface MHC I, ICAM-1, and ICAM-2 molecules [25], numerous previous studies have reported mitochondrial dysfunction in HTLV-1 infected patients [26]. One study reported differences in the number and morphology of mitochondria within myofibers of HTLV-1-infected and non-infected myositis patients [27]. HTLV-1 infection may alter the myofiber intracellular environment. Because HTLV-1 infection and nemaline myopathy are rare worldwide, it is difficult to determine their association epidemiologically.

To the best of our knowledge, this is the first report of a case of SLONM associated with HTLV-1 infection. Whether HTLV-1 causes chronic muscle disease remains unclear, and further research is needed on the relationship between retroviruses and muscle diseases.

## Data Availability

Data sharing is not applicable to this article as no datasets were generated or analyzed during the current study.

## Abbreviations

HTLV-1: Human T-lymphotropic virus type 1
HAM/TSP: HTLV-1-associated myelopathy/tropical spastic paraparesis
SLONM: Sporadic late-onset nemaline myopathy
MGUS: Monoclonal gammopathy of unknown significance
HIV: Human immunodeficiency virus
CSF: Cerebrospinal fluid
SEP: Somatosensory evoked potential

## References

[1] Engel AG (1966). Late-onset rod myopathy (a new syndrome?): light and electron microscopic observations in two cases. Mayo Clin Proc.

[2] Chahin N, Selcen D, Engel AG (2005). Sporadic late onset nemaline myopathy. Neurology.

[3] Suzuki M, Shimizu Y, Takeuchi M, Kobayashi M, Iwata M, Uchiyama S (2012). Sporadic late-onset nemaline myopathy in a patient with primary Sjogren’s syndrome. J Neurol.

[4] Cao L, Wang Y, Liu X, Hu Y, Li N, Qiu G, Luo Y, Li W (2016). Adult-onset nemaline myopathy coexisting with myasthenia gravis: a case report. Medicine (Baltimore).

[5] Hindocha A, Klimiuk P, Roberts M, Pal P, Evangelista T, Lochmuller H, Chinoy H (2017). Co-presentation of adult-onset systemic lupus erythematosus and nemaline myopathy. Rheumatology (Oxford).

[6] Dalakas MC, Pezeshkpour GH, Flaherty M (1987). Progressive nemaline (rod) myopathy associated with HIV infection. N Engl J Med.

[7] Maytal J, Horowitz S, Lipper S, Poiesz B, Wang CY, Siegal FP (1993). Progressive nemaline rod myopathy in a woman coinfected with HIV-1 and HTLV-2. Mt Sinai J Med.

[8] Schnitzler LJ, Schreckenbach T, Nadaj-Pakleza A, Stenzel W, Rushing EJ, Van Damme P, Ferbert A, Petri S, Hartmann C, Bornemann A (2017). Sporadic late-onset nemaline myopathy: clinico-pathological characteristics and review of 76 cases. Orphanet J Rare Dis.

[9] Uruha A, Benveniste O (2017). Sporadic late-onset nemaline myopathy with monoclonal gammopathy of undetermined significance. Curr Opin Neurol.

[10] Kumutpongpanich T, Owattanapanich W, Tanboon J, Nishino I, Boonyapisit K (2018). Sporadic late-onset nemaline myopathy with monoclonal gammopathy of undetermined significance (SLONM-MGUS): An alternative treatment using cyclophosphamide-thalidomide-dexamethasone (CTD) regimen. Neuromuscul Disord.

[11] Milone M, Katz A, Amato AA, Soderland CA, Segarceanu M, Young NP, Jones HR (2010). Sporadic late onset nemaline myopathy responsive to IVIg and immunotherapy. Muscle Nerve.

[12] Uchiyama T, Yodoi J, Sagawa K, Takatsuki K, Uchino H (1977). Adult T-cell leukemia: clinical and hematologic features of 16 cases. Blood.

[13] Osame M, Usuku K, Izumo S, Ijichi N, Amitani H, Igata A, Matsumoto M, Tara M (1986). HTLV-I associated myelopathy, a new clinical entity. Lancet.

[14] Nakao K, Ohba N (1993). Clinical features of HTLV-I associated uveitis. Br J Ophthalmol.

[15] Sato T, Coler-Reilly A, Utsunomiya A, Araya N, Yagishita N, Ando H, Yamauchi J, Inoue E, Ueno T, Hasegawa Y (2013). CSF CXCL10, CXCL9, and neopterin as candidate prognostic biomarkers for HTLV-1-associated myelopathy/tropical spastic paraparesis. PLoS Negl Trop Dis.

[16] Arimura K, Arimura Y, Moritoyo H, Tokimura Y, Takenaga S, Sonoda Y, Yamanaka H, Nakagawa M, Izumo S, Osame M (1995). How helpful is thoracic paraspinal EMG in HAM/TSP?. Muscle Nerve.

[17] Moritoyo H, Arimura K, Arimura Y, Tokimura Y, Rosales R, Osame M (1996). Study of lower limb somatosensory evoked potentials in 96 cases of HTLV-I-associated myelopathy/tropical spastic paraparesis. J Neurol Sci.

[18] Osame M, WA B (1990). Review of WHO Kagoshima meeting and diagnostic guidelines for HAM/TSP. Human retrovirology: HTLVedn.

[19] Dalakas MC (2006). Sporadic inclusion body myositis–diagnosis, pathogenesis and therapeutic strategies. Nat Clin Pract Neurol.

[20] Cupler EJ, Leon-Monzon M, Miller J, Semino-Mora C, Anderson TL, Dalakas MC (1996). Inclusion body myositis in HIV-1 and HTLV-1 infected patients. Brain.

[21] Ozden S, Gessain A, Gout O, Mikol J (2001). Sporadic inclusion body myositis in a patient with human T cell leukemia virus type 1-associated myelopathy. Clin Infect Dis.

[22] Matsuura E, Kubota R, Saito M, Suehara M, Matsuzaki T, Arimura K, Osame M, Izumo S (2006). Visualization of HTLV-I Tax-specific cytotoxic T lymphocytes in the central nervous system of HTLV-I-associated myelopathy. J Neuroimmunol.

[23] Gilbert DT, Morgan O, Smikle MF, Simeon D, Barton EN (2001). HTLV-1 associated polymyositis in Jamaica. Acta Neurol Scand.

[24] Gabbai AA, Wiley CA, Oliveira AS, Smith R, Schmidt B, Nobrega JA, Bordin JO, Roman GC (1994). Skeletal muscle involvement in tropical spastic paraparesis/HTLV-1-associated myelopathy. Muscle Nerve.

[25] Banerjee P, Feuer G, Barker E (2007). Human T-cell leukemia virus type 1 (HTLV-1) p12I down-modulates ICAM-1 and -2 and reduces adherence of natural killer cells, thereby protecting HTLV-1-infected primary CD4+ T cells from autologous natural killer cell-mediated cytotoxicity despite the reduction of major histocompatibility complex class I molecules on infected cells. J Virol.

[26] Ciminale V, Zotti L, D'Agostino DM, Ferro T, Casareto L, Franchini G, Bernardi P, Chieco-Bianchi L (1999). Mitochondrial targeting of the p13II protein coded by the x-II ORF of human T-cell leukemia/lymphotropic virus type I (HTLV-I). Oncogene.

[27] Abdullah HM, Higuchi I, Kubota R, Matsuura E, Hashiguchi A, Abdelbary NH, Inamori Y, Takashima H, Izumo S (2011). Histopathological differences between human T-lymphotropic virus type 1-positive and human T-lymphotropic virus type 1-negative polymyositis. Clin Exp Neuroimmunol.

